# Identification and Immobilization of an Invertase With High Specific Activity and Sucrose Tolerance Ability of *Gongronella* sp. w5 for High Fructose Syrup Preparation

**DOI:** 10.3389/fmicb.2020.00633

**Published:** 2020-04-09

**Authors:** Gang Zhou, Can Peng, Xiaosa Liu, Fei Chang, Yazhong Xiao, Juanjuan Liu, Zemin Fang

**Affiliations:** ^1^School of Life Sciences, Anhui University, Hefei, China; ^2^Anhui Key Laboratory of Modern Biomanufacturing, Hefei, China; ^3^Anhui Provincial Engineering Technology Research Center of Microorganisms and Biocatalysis, Hefei, China

**Keywords:** invertase, expression, immobilization, high fructose syrup, *Gongronella* sp.

## Abstract

Invertases catalyze the hydrolysis of sucrose into fructose and glucose and can be employed as an alternative in producing high fructose syrup. In this study, we reported the heterologous expression of an invertase gene (*GspInv*) of *Gongronella* sp. w5 in *Komagataella pastoris*. GspInv activity reached 147.6 ± 0.4 U/mL after 5 days of methanol induction. GspInv is invertase with a high specific activity of 2,776.1 ± 124.2 U/mg toward sucrose. GspInv showed high tolerance to sucrose (*IC*_5__0_ = 1.2 M), glucose (*IC*_5__0_ > 2 M), fructose (*IC*_5__0_ = 1.5 M), and a variety of metal ions that make it an ideal candidate for high fructose syrup production. A carbohydrate-binding module was sequence-optimized and fused to the N-terminus of GspInv. The fusion protein had the highest immobilization efficiency at room temperature within 1 h adsorption, with 1 g of cellulose absorption up to 8,000 U protein. The cellulose-immobilized fusion protein retained the unique properties of GspInv. When applied in high fructose syrup preparation by using 1 M sucrose as the substrate, the sucrose conversion efficiency of the fused protein remained at approximately 95% after 50 h of continuous hydrolysis on a packed bed reactor. The fused protein can also hydrolyze completely the sucrose in sugarcane molasses. Our results suggest that GspInv is an unusual invertase and a promising candidate for high fructose syrup preparation.

## Introduction

High fructose syrup (HFS) is a major nutritive sweetener presented as a mixture of glucose and fructose (Singh et al., 2018a). In modern food and beverage industries, HFS has replaced sucrose as an alternative sweetener because of its many advantages (Rippe, 2014). For example, HFS is easier to store than sucrose because of its high osmotic pressure and anti-drying properties (Singh et al., 2018a). Furthermore, HFS is crystallization-controlled and easy to dissolve, making it flexible for many applications, such as seasoning and baking (Zhang et al., 2004). HFS can be divided into three categories according to fructose content: HFS-90 (90% fructose and 10% glucose), HFS-55 (55% fructose and 45% glucose), and HFS-42 (42% fructose and 58% glucose). In general, HFS-55 is more suitable for industrial applications than HFS-42 it has better flavor, sweetness, and other benefits (Fatourehchi et al., 2014; Neifar et al., 2019).

Commercially, HFS is obtained from cornstarch through multi-enzymatic hydrolysis and transformation in four steps, including (a) enzymatic liquefaction or partial hydrolysis of starch with α-amylase, (b) conversion of liquefied starch into dextrose hydrolyzate by employing amylo-glucosidase, (c) isomerization of dextrose to fructose using isomerase, and (d) refinement of the final fructose product (Singh et al., 2018a). Ultra-filtration and crystallization steps, such as activated carbon decolorization, ion exchange, chromatographic separation, and evaporation, are necessary to obtain high-purity fructose (Guthrie and Morton, 2000; Chen et al., 2016). All these complicated steps address related problems smoothly because the conventional HFS production approach is well-established commercially. However, certain drawbacks, including low conversion rate, low separation efficiency, labor-intensive preparation, poor enzyme technology, and low product yields, increase production costs (Wang et al., 2016). Hence, a convenient and cost-effective technique for HFS production needs to be developed.

Invertases (EC 3.2.1.26) are enzymes that catalyze the hydrolysis of sucrose into equimolar concentrations of glucose and fructose (inverted syrup) (Kotwal and Shankar, 2009). One of the most significant applications of invertase lies in the production of inverted syrup that can be used directly as HFS or as a substrate to obtain pure crystalline fructose (Kotwal and Shankar, 2009; Lima et al., 2011). However, the use of commercially available invertases to hydrolyze sucrose is costly because of the many drawbacks from substrate and enzyme aspects. For instance, the price of commercial invertase is high (Torres-Acosta et al., 2018). Meanwhile, most invertases are inhibited by the substrate sucrose and the end-product glucose and fructose (Sakakibara et al., 1996; Rashad and Nooman, 2009; Resa et al., 2009). For example, sucrose concentrations higher than 50 mM cause the yield to diminish sharply when commercially available invertase is used as the catalyst (Tomotani and Vitolo, 2007). Moreover, fructose and glucose are non-competitive inhibitors of invertase activities at high concentrations (Isla et al., 1999). Invert syrup production from sugarcane or beet sucrose is unavailable economically. Molasses (a by-product from sugarcane processing) (Deng et al., 2008) is used as the alternative substrate because it is about 10-times lower in price than the sucrose (Khatiwada et al., 2016; Gabisa et al., 2019) and is rich in sucrose and glucose (30–50%, v/v) and metal ions (Liu et al., 2008; Xia et al., 2016). Therefore, finding novel invertases that fit the characteristics of molasses, such as tolerance to high sucrose content and metal ions, and low-cost preparation is beneficial economically (Palai et al., 2014; Eskandarloo and Abbaspourrad, 2018; Mohammadi et al., 2018).

*Gongronella* sp. w5 is a soil-borne fungus that prefers sucrose as its carbon source (Hu et al., 2018). Previously, a deduced cytoplasmic glycoside hydrolase family 32 (GH32) invertase (named GspInv), with no significant sequence identity with well-characterized fungal invertases, was predicted in the genome (Dong et al., 2018). In the present study, GspInv was cloned and expressed in *Komagataella pastoris*. Our results showed GspInv is an unusual invertase suitable for HFS production. Furthermore, HFS production cost was reduced by fusing a sequence-optimized carbohydrate-binding module (CBM) (Oliveira et al., 2015) to the N-terminal of GspInv to facilitate the purification and immobilization of GspInv. The fused protein showed high efficiency in sucrose and molasses hydrolysis. As such, GspInv and its derivates have remarkable application potential in HFS production.

## Materials and Methods

### Strains, Plasmid, and Reagents

*Gongronella* sp. w5 was obtained from China Center for Type Culture Collection (No. AF2012004) and cultured on potato dextrose agar slants at 4°C. *K. pastoris* GS115 and the expression vector pPIC9K were purchased from Invitrogen (Carlsbad, CA, United States). Yeast extract peptone dextrose medium, minimal dextrose medium (MD), buffered minimal glycerol, and buffered minimal glycerol yeast medium (BMGY) were prepared in accordance with the manual of the EasySelect *Komagataella* (*Pichia*) Expression kit (Invitrogen). All other chemicals and reagents were of analytical grade unless otherwise indicated.

### Cloning, Expression, and Purification of Recombinant GspInv and Its Derivatives

Five fungal plugs of *Gongronella* sp. w5 with a diameter of 5 mm were grown at 37°C and 120 rpm in SAHX medium in accordance with the method of Hu et al. (2018). After growing for 2 days, the mycelia were collected and grounded with a mortar and pestle in the presence of liquid nitrogen. Total RNA was extracted using TRIzol reagent according to the manufacturer’s instruction (TaKaRa, Dalian, China), followed by RNase-free DNase digestion (Promega, Beijing, China). The first-strand cDNA was synthesized using reverse transcriptase and a primer pair of *GspInvF* CCTAGGATGGTGCTTGCTGATCCT (*Bln*I site underlined) and *GspInvR* GCGGCCGCTCAAGGGCGATTGAACG (*Not*I site underlined) in accordance with the manufacturer’s protocol (TaKaRa). GspInv cDNA was amplified by PCR by using the same primer pair of *GspInvF* and *GspInvR*. The cloned cDNA was digested further with *Eco*RI and *Not*I, ligated into the similarly digested expression plasmid pPIC9K, and linearized and transformed into *K. pastoris* GS115. The electroporated cells were plated onto MD agar plates to select the His^+^ transformants. Some His^+^ transformants were selected randomly and grown on the BMGY liquid medium at 28°C for 2 days.

GspInv was purified and immobilized onto cellulose through fusing a CBM_24_ from *Clostridium thermocellum* (GenBank No. HF912724.1) to GspInv (Chang et al., 2018). In brief, the CBM_24_ gene was codon-optimized in accordance with the *K. pastoris* codon bias and synthesized by Sangon Biotech (Shanghai, China). It was fused to the N- or C-terminus of *GspInv* by using overlap extension PCR (Chang et al., 2018). Given that CBM_24_ contains three potential glycosylation sites at 11, 65, and 121 in the amino acid sequence, the amino acids at these sites were mutated to aspartic acid to obtain CBM_24_DG (DG stands for deglycosylation). The mutated sequence was fused further to the N- or C-terminus of the GspInv by using overlap extension PCR. Four plasmids, namely, CBM_24_-GspInv, CBM_24_DG-GspInv, GspInv-CBM_24_, and GspInv-CBM_24_DG, were constructed after digesting the four sequences with *Not*I and *Avr*II and inserting into the *K. pastoris* expression vector pPIC9K. They were transformed individually into *K. pastoris* cells and screened as described above.

The proteins were expressed by cultivating *K. pastoris* GS115 host cells carrying GspInv, CBM_24_-GspInv, CBM_24_DG-GspInv, GspInv-CBM_24_, and GspInv-CBM_24_DG separately in 500 mL flasks containing 100 mL of BMGY medium at 28°C and shaken at 220 rpm. When the cell density of *OD*_600_ reached 2.0–6.0, cells were harvested and resuspended in 150 mL of BMMY medium in 500 mL flasks at a final cell density of *OD*_600_ = 1.0. The cultures were incubated at 28°C and shaken at 220 rpm with a daily addition of 0.5% (v/v) methanol to induce extracellular GspInv production. Samples were withdrawn every 24 h to determine the invertase activity and the biomass.

The aqueous solution was centrifuged at 20,000 × *g* for 30 min and concentrated at 4°C through ultrafiltration using a Minitan ultrafiltration system with a low-binding regenerated cellulose membrane (Millipore, Bedford, MA, United States). The heterologously expressed protein from *K. pastoris* GS115 culture broth was purified by centrifuging the concentrate at 20,000 × *g* for 30 min, and the supernatant was then dialyzed overnight against the citrate-phosphate buffer (50 mM, pH 5.5), followed by centrifugation as described previously. Then the supernatant was applied to a DEAE-Sepharose FF column (10 mm × 200 mm, Amersham Pharmacia, Uppsala, Sweden) pre-equilibrated with citrate-phosphate buffer. The column was eluted with a linear gradient of (NH_4_)_2_SO_4_ (0–0.5 M in a citrate-phosphate buffer, with a flow rate of 1.0 mL min^–1^).

### Invertase Activity Assay

Protein samples were diluted in a suitable volume of citrate-phosphate buffer (50 mM, pH 5.0). Invertase activities were measured in 1 mL of reaction mixtures containing 20 μL purified enzyme, 50 mM citrate-phosphate buffer (pH 5.0), and 200 mM sucrose, and incubated at 45°C for 5 min. The reaction was terminated by heating the assay mixture at 100°C for 5 min. The released glucose and fructose were measured using the 3,5-dinitrosalicylic acid method (Ashwell, 1957). The unit (U) of invertase activity was defined as the amount of enzyme required to hydrolyze 1 μmol of sucrose per min under assay conditions.

### Biochemical Characterization of Recombinant Proteins

The protein concentration was assayed using the Bradford method at 595 nm, with bovine serum albumin as the standard (Sangon Biotech). The homogeneity of GspInv and its derivatives was determined by 15% sodium dodecyl sulfate polyacrylamide gel electrophoresis (SDS-PAGE) and stained with Coomassie brilliant blue R250. To verify that the protein in the gel was the recombinant GspInv, SDS-PAGE gel was washed with 50 mM citrate-phosphate buffer at pH 5.0 for 1 h to remove SDS. Then, it was incubated in an acetate-phosphate buffer (50 mM, pH 5.0) containing 200 mM sucrose at 45°C for 30 min, and actively stained using 100 mM NaOH solution containing 0.2% triphenyl tetrazolium chloride after the sucrose solution was removed (Van et al., 2013).

The effect of pH on enzymatic activity was determined at 45°C in 50 mM citrate-phosphate buffer (pH 3.5–6.5). The effect of temperature on the enzymatic activity was determined at pH 5.0 and temperatures ranging from 30–60°C. The enzyme stabilities against pH and temperature were determined by incubating proteins at various temperatures and different pH values. The residual activities were determined as mentioned above. All experiments were performed in triplicate.

The effects of metal ions including Mg^2+^, Ba^2+^, Ca^2+^, Co^2+^, Ni^2+^, Mn^2+^, Cu^2+^, and Fe^3+^, on GspInv activity were investigated in the presence of each ion at different concentrations (1, 5, and 10 mM). The enzyme assays were carried out at pH 5.0 and 45°C.

### Kinetic Analysis

The appropriate concentration of GspInv or its derivatives was utilized under the optimum conditions to determine the kinetic parameters (*K*_m_, *V*_max_, and *k*_cat_/*K*_m_). The reaction was carried out by incubating the enzyme in 50 mM citrate-phosphate buffer (pH 5.0) containing sucrose at a concentration range of 1 mM–2 M at 45°C for 5 min. Released glucose was quantified by using the glucose oxidase method (Rongsheng Biotech, Shanghai, China). The kinetic constants and their corresponding errors were calculated by fitting the measured rate to the Michaelis–Menten equation using the computer program Origin 8.0 (*n* = 9).

### Effects of Mono-and Di-Sugars on Invertase Activity

The effect of sucrose on GspInv and its derivates activity was determined using 0.01–2 M sucrose as the substrate. The feedback inhibition of fructose and glucose on invertase activity was determined through the use of 0, 0.2, 0.4, 0.6, 0.8, 1, 1.2, 1.5, and 2.0 M glucose or fructose added to the enzymatic reaction systems. The content of the glucose in the final product after the reaction was measured via the glucose oxidase method. The fructose concentration was determined using high-performance liquid chromatography (HPLC). Briefly, 20 μL of samples were withdrawn at different time intervals and analyzed at 50°C by using a TSKgel Amide-80 column (4.6 mm × 250 mm, 5 μm, Tosoh Corporation, Kyoto, Japan) and an evaporative light-scattering detector 2424 (Waters, United States). The eluting solution was acetonitrile: water (70:30, v/v).

### Immobilization of Enzyme on Cellulose

The fermentation supernatant containing CBM_24_DG-GspInv was withdrawn by centrifuging the cultures at 12,000 × *g* for 10 min and used directly for purification and immobilization. The microcrystalline cellulose was added into the supernatant at a ratio of 1:20 (g/mL) and incubated at 25°C for 4 h in a shaker at 60 rpm/min. The samples were withdrawn at intervals of 0.5 h during the process and centrifuged at 5,000 × *g* to separate the supernatant and precipitate. The precipitate was then washed three times with an equal volume of citrate-phosphate buffer (50 mM, pH 5.0) and stored in this buffer. The residual invertase activity in the supernatant and the invertase activity on immobilized cellulose were separately tested. The immobilization efficiency of cellulose was calculated.

### Reusability of Immobilized Invertase

For the continuous hydrolysis of sucrose, a packed bed reactor (PBR) was prepared by using 10 g of cellulose-CBM_24_DG-GspInv (82,000 U) with a fixed bed height of 10 cm. The reactor was placed in a thermo-stated oven (Notting Scientific Equipment, Hangzhou, China) at 35°C to ensure constant reaction temperature. Then, the sucrose solution (1, 1.2, 1.5, or 2 M prepared in a citrate-phosphate buffer, pH 5.0) was fed into the base of the reactor by using a pump (Notting Scientific Equipment) at a flow rate of 2.5 mL/min for 5, 10, and 15 h, respectively. The eluent was withdrawn and the concentrations of sucrose, glucose, and fructose analyzed using HPLC as described above. The sucrose hydrolysis efficiency was then calculated.

The PBR containing cellulose-CBM_24_DG-GspInv prepared as described above was loaded continuously with 1 M sucrose for 50 h at a flow rate of 2.5 mL/min to determine the reusability of cellulose-CBM_24_DG-GspInv. The eluent was withdrawn every 5 h, and the concentrations of sucrose, glucose, and fructose analyzed.

When molasses was employed as the substrate, the crude molasses (Xuanbo sugar-refinery, Guangxi, China) contains 40% (w/v) sucrose, 13.2% reduced sugars (glucose and fructose), 2.3% other carbohydrates, 9.1% ash, 3.9% salt, and 8.4% mineral substance was diluted with citrate-phosphate buffer (50 mM, pH 5.0) to obtain 20% sucrose concentration and centrifuged at 8,000 × *g* for 5 min to remove impurities. Then, 5 mg/L polyacrylamide was added into the liquid molasses, which was incubated at 85°C for 10 min, followed by centrifugation at 8,000 × *g* for 5 min to remove the precipitates. The supernatant was loaded to the PBR for 5 h at a flow rate of 2.5 mL/min. The eluent was withdrawn every 1 h and the concentrations of sucrose, glucose, and fructose analyzed.

### Bioinformatics Analysis

The sequence similarity search of GspInv was performed using Blast at UniProt^1^. The module structure of the enzyme was analyzed with a simple modular architecture research tool SMART^2^. Multiple sequence alignment of GspInv with other related invertase sequences was performed using Clustal X 2.0 and Phylogeny fr^3^. The phylogenetic tree was constructed using MEGA 7.

## Results and Discussion

### GspInv Cloning and Sequence Analysis

The full-length GspInv cDNA was amplified successfully from the total RNA extracted from *Gongronella* sp. w5 mycelia using sucrose as the carbon source, with primers binding specifically to upstream and downstream of the cDNA coding sequence of GspInv deduced from the genome (Dong et al., 2018). The cloned GspInv cDNA was 1,761 bp in length. Compared to the 1,822 bp of its gene sequence, *GspInv* harbors only a 70 bp intron located between nucleotides 208 and 277. GspInv comprises 586 amino acids in length, with a theoretical molecular weight of 65.4 kDa and an isoelectric point of 4.89. The GspInv amino acid sequence shares the highest identities of 62.4 and 44% with a glycosyl hydrolase (ORZ22493.1) and a glycoside hydrolase family 32 protein (XP_018284693) deduced from the genome of *Absidia repens* and *Phycomyces blakesleeanus* NRRL 1555 and has not been characterized biochemically. Further analysis showed that GspInv has sequence identities of less than 37% to hypothetical proteins deduced from the genome sequences (Supplementary Table S1). These results suggested that GspInv is a novel protein with little information gained on its biochemical characteristics.

**FIGURE 1:**
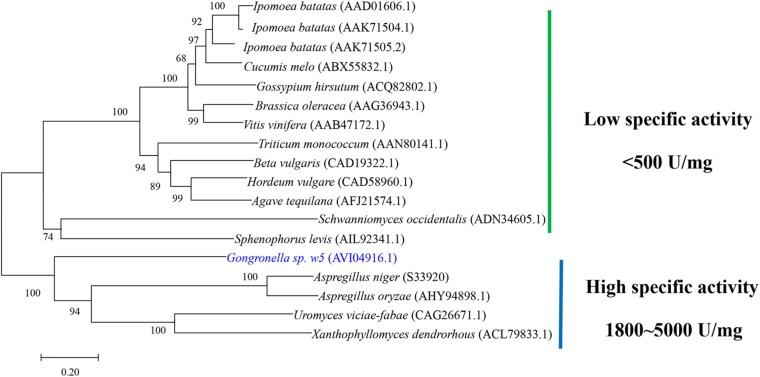
Neighbor-joining phylogeny of GspInv with experimentally characterized fungal GH32 invertases. Sequences are retrieved from GenBank database. GenBank protein accession numbers are given in parentheses. Bootstrap support values based on 1000 replicates are given as percentages above individual branches.

No signal peptide was predicted at the N-terminal of GspInv by using SingalP 5.0, which suggests that GspInv is an intracellular protein. This result is consistent with the invertase activity that can only be detected in the intracellular proteome of *Gongronella* sp. w5 (Hu et al., 2018). Module analysis suggested that GspInv is a GH32 protein carrying Pfam domain signatures, with accession numbers PF00251 (E value 3e-49) and PF08244 (E value 3.22e-25), which are characteristics of GH32 family protein (Finn et al., 2017). The alignment of the GspInv amino acid sequence with other characterized invertases revealed the GspInv contains conserved motifs of GH32 invertases (Supplementary Figure S1). Based on their alignments, residues D46, D173, and E251 of GspInv were identified as the nucleophile, transition-state stabilizer, and general acid/base catalyst, respectively (Supplementary Figure S1; Pons et al., 2004; Ramírez-Escudero et al., 2016). Trollope et al. (2015) reported that GH32 invertases can be divided into two groups, including that with low- and high-level fructooligosaccharide synthesis abilities based on the conserved motifs unique to each group. According to their prediction, GspInv belongs to the invertase with low-level fructooligosaccharide synthesis ability. Many GH32 members have been well characterized experimentally. In addition to sucrose hydrolysis ability, some invertases exhibit transferase activity at high substrate concentrations, resulting in the formation of fructooligosaccharides by transferring successive fructose units to sucrose (Trollope et al., 2015). The invertase with low-level fructooligosaccharide synthesis ability will facilitate the preparation of HFS using high concentrations of sucrose as the substrate. Moreover, phylogenetic tree analysis suggested that GspInv belongs to the clade of invertases with high specific activity (Figure 1), and as such, GspInv may have application potential in modern industries.

### Expression of GspInv in *K. pastoris* and Purification

**FIGURE 2:**
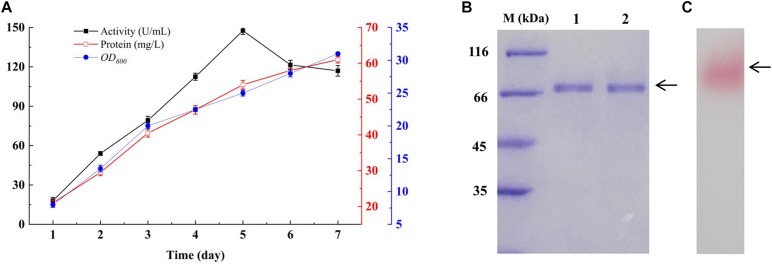
Expression of GspInv in *K. pastoris*. **(A)** Time course of invertase activity, protein content, and *K. pastoris* concentration (*OD*_600_) in the extracellular medium of *K. pastoris* cultures producing recombinant GspInv. The data presented are the average values from triplicate cultures (repeats of experiments gave similar results). **(B)** Coomassie brilliant blue R250 stained 15% SDS-PAGE gel of GspInv in crude culture supernatant (lane 1) and after ion-exchange chromatography (lane 2). **(C)** Zymogram gel of GspInv in crude culture supernatant.

GspInv was expressed in *K. pastoris* under the control of the inducible promoter AOX1 and with the α-factor signal sequence of the vector pPIC9K for secreted expression. The highest invertase activity of 147.6 ± 0.4 U/mL was obtained after induction with methanol for 5 days and using sucrose as the substrate. The extracellular soluble protein content was determined as 54.1 ± 0.7 mg/L (Figure 2A). Given that the specific activity of GspInv is 2,776.1 ± 124.2 U/mg as determined below, approximately 95% (>51 mg) of the total protein in the culture supernatant was GspInv on the 5th day. As such, only one band with an apparent molecular weight of approximately 67 kDa was presented on SDS-PAGE gel and the purity of the protein was similar to that after ion-exchange chromatography (Figure 2B). After active staining, the color of the band changed to pink, indicating that the protein was GspInv (Figure 2C; Van et al., 2013). MALDI-TOF-MS/MS identification of the peptide also suggested the only band on the gel was GspInv (Supplementary Table S2).

*K. pastoris* is considered one of the best hosts for invertase expression. Invertases from several strains, such as *Zymomonas mobilis* and *Aspergillus niger*, have been expressed heterologously in *K. pastoris* (Veana et al., 2014; Pérez de los Santos et al., 2016). The GspInv productivity was considerably higher than those of most invertases expressed heterologously in *K. pastoris* (Supplementary Table S3; Hsieh et al., 2006; Plascencia-Espinosa et al., 2014). Furthermore, GspInv is electrophoretically homogenous on an SDS-PAGE gel after ultrafiltration, which means no additional purification step is necessary to purify GspInv and decrease production costs. This result is also different from most invertases with purification steps that require ultrafiltration and complicated chromatographic techniques that result in more than 50% activity loss (Huang et al., 2003; Wang et al., 2005; Hsieh et al., 2006; Veana et al., 2014; Pérez de los Santos et al., 2016; Supplementary Table S3). The complicated purification steps of these invertases cause the large-scale enzymatic hydrolyzing reactions to be impractical economically.

### GspInv Characterization

**FIGURE 3:**
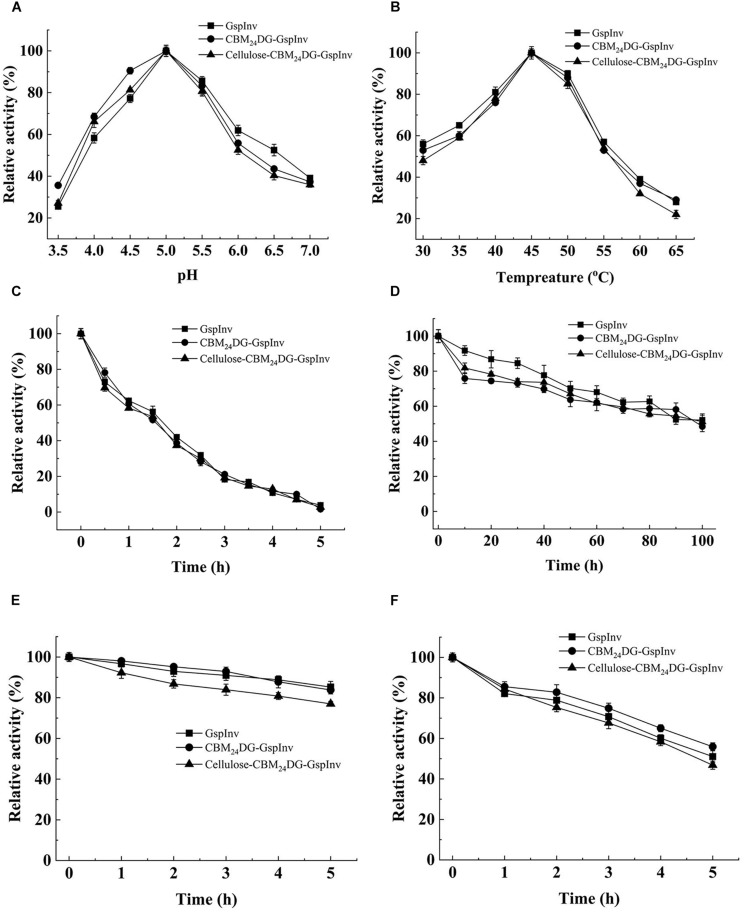
Effects of pH and temperature on the activity and stability of GspInv, CBM_24_DG-GspInv, and cellulose-CBM_24_DG-GspInv. **(A)** pH optimum. Samples were incubated at 45°C. **(B)** Temperature optimum. Samples were incubated at pH 5.0. **(C,D)** Thermostability at 45°C and 40°C, respectively. Samples were incubated at pH 5.0. **(E,F)** pH stability at pH 5.5 and pH 6.0, respectively. Samples were incubated at 4°C. Standard deviations and values were calculated from triplicate technical repeats of measurements.

The optimum pH of the purified GspInv was at pH 5.0. It showed more than 50% of its activity at a pH range of 4.0–6.5 (Figure 3A) and decreased sharply at pH 3.5 and above 6.5 when evaluated using sucrose as the substrate. The optimum temperature of GspInv was 45°C. More than 50% of the protein activity was shown when tested at temperatures between 30 and 55°C (Figure 3B). The optimum temperature and pH of GspInv were similar to most of the invertases from other fungi, with optimum temperature and pH of 40–70°C and 4.5–6.5, respectively (Acosta et al., 2000; Cai et al., 2014; Nadeem et al., 2015).

The thermostability of GspInv was assayed at the optimum pH 5.0. It retained approximately 65% of its original activity after incubation at 45°C for 1 h (Figure 3C). In comparison, GspInv retained 50% of the original activity after incubation at 40°C for 100 h (Figure 3D). GspInv was stable at pH 5.5. After incubation at pH 5.5 and 4°C for 5 h, the protein retained approximately 85% of its original activity (Figure 3E). GspInv retained 55% of its activity after 5 h at pH 6.0 (Figure 3F). pH and thermal stabilities are considered commercially as profitable features of an enzyme. Enzyme-catalyzed reactions operating at moderate temperatures and weak acidic pH reduce costs on energy and equipment (Singh et al., 2018b).

The effects of metal ions on GspInv activity were assayed. Invertase activity increased to 109.7 ± 4.4% (10 mM), 120.9 ± 3.7% (10 mM), and 153.8 ± 3.6% (1 mM) in the presence of Ba^2+^, Ca^2+^, and Mn^2+^, respectively. The presence of metal ion Mn^2+^ increasing the enzymatic activity has been reported for several invertases. For example, it increased the INVA and INVB activities by 80 and 20%, respectively (Pérez de los Santos et al., 2016). This activating effect has been observed for the invertase from the yeast *Candida guilliermondii* (Plascencia-Espinosa et al., 2014). Mn^2+^ enhanced its activity by up to 277% for invertase from *Anthene phoenicis* (Rustiguel et al., 2015). GspInv retained 82.2 ± 6.9, 72.8 ± 11.1, and 81.3 ± 2.4% of its original activities in the presence of 10 mM Mg^2+^, Co^2+^, and Fe^3+^, respectively. The tolerance of GspInv on these ions suggested it can hydrolyze substances such as molasses rich in sucrose but contain various ions. In comparison, ions including Ni^2+^ and Cu^2+^ showed inhibitory effects on the activity under all tested concentrations (1, 5, and 10 mM). GspInv activities were reduced to 57.9 ± 2.3 and 16.7 ± 1.4% at 10 mM of Ni^2+^ and Cu^2+^ (Table 1). This finding suggests the presence of thiol groups or His residues that are important for enzyme activity. Co^2+^, Ni^2+^, and Cu^2+^ may coordinate with His residues on protein groups and produce conformational changes in the protein structure (Pérez de los Santos et al., 2016). Furthermore, Cu^2+^ oxidizes cysteine residues in the protein and cause structural changes and alteration in the protein activity.

**TABLE 1:** Effects of metal ions on GspInv activity.

Metal ions	1 mM	5 mM	10 mM
None	100.0	100.0	100.0
Mg^2+^	102.6 ± 1.8	90.7 ± 8.5	82.2 ± 6.9
Ba^2+^	106.7 ± 11.1	108.1 ± 3.9	109.7 ± 4.4
Ca^2+^	111.9 ± 4.9	112.7 ± 3.0	120.9 ± 3.7
Co^2+^	102.1 ± 6.2	85.1 ± 7.6	72.8 ± 11.1
Ni^2+^	92.4 ± 1.4	78.7 ± 4.2	57.9 ± 2.3
Mn^2+^	153.8 ± 3.6	143.2 ± 1.6	113.3 ± 17.2
Cu^2+^	52.1 ± 2.3	22.3 ± 1.9	16.7 ± 1.4
Fe^3+^	88.6 ± 4.6	89.2 ± 3.1	81.3 ± 2.4

*Sulfates are used as the target mental ion donators. Invertase activity was determined at 45°C in citrate-phosphate buffer (50 mM, pH 5.0) using sucrose as the substrate. The data presented are the average values from triplicate technical repeats of measurements.*

The substrate specificity and action mode of GspInv were investigated by incubating the enzyme with sucrose, trehalose, cellobiose, maltose, isomaltose, raffinose, melezitose, stachyose, and inulin at pH 5.0 and 45°C. Similar to most of yeast invertases, which are active only against sucrose and raffinose (Plascencia-Espinosa et al., 2014), GspInv released fructose from sucrose and raffinose and did not exhibit hydrolysis activity toward other substrates (Supplementary Table S4), suggesting that GspInv is invertase (Zhang et al., 2015). The kinetic constants of GspInv on sucrose were tested at optimal conditions. The values of the kinetic parameters *K*_m_, *k*_cat_, and *k*_cat_/*K*_m_ were 8.7 ± 1.1 mM, 5,100 ± 10.8 s^–1^, and 595 ± 76.6 mM^–1^ s^–1^, respectively. The *K*_m_ value of GspInv falls at the lower end of the *K*_m_ values of 9.1–61.2 mM reported for most invertases (Table 2). This result suggests a relatively higher affinity for sucrose than most of invertases (Pérez et al., 2001; Nadeem et al., 2009; Zhou et al., 2016). In accordance with the phylogenetic analysis (Figure 1), the specific activities of GspInv on sucrose and raffinose were 2,776.1 ± 124.2 and 2,098.7 ± 123.6 U/mg, respectively (Supplementary Table S4), considerably higher than that of most invertases, such as that from *Aspergillus foetidus* (257.2 U/mg) (Rehm et al., 1998; Table 2).

**TABLE 2:** Comparison of the biochemical characteristics and kinetic constants of GspInv with characterized invertases.

Source	Optimum		Specific activity (U/mg)	Kinetic parameters		References
	pH	Temp (°C)		*K*_m_ (mM)	*k*_cat_ (s^–1^)	
*A. niger*	5.0–6.5	65–70	NR	4 ± 0.5	NR	Veana et al., 2014
*S. cerevisiae*	3.5–5.5	60	1590	NR	NR	Acosta et al., 2000
*A. niger* (SucB)	5.0	40	NR	2.0 ± 0.2	NR	Goosen et al., 2007
*Bacillus* sp. HJ14	8	30	2400	62.9	746.2	Zhou et al., 2016
*Pichia anomala*	4.0–6.5	38	1482	16	NR	Pérez et al., 2001
*Elsholtzia haichowensis*	5	70	NR	2.68 ± 0.1	NR	Cai et al., 2014
*Aspergillus foetidus*	5.6	37	257.2	NR	NR	Rehm et al., 1998
*Gongronella* sp. w5	5	45	2776 ± 124	8.7 ± 1.1	5100	This study

*NR, not reported.*

The bioinformatics and biochemical data presented in this study support the conclusion that GspInv is invertase. Fungi are a rich source of GH32 invertases. However, no invertase from genus *Gongronella* has been reported, although previous studies have shown that fungi from genus *Gongronella*, such as *Gongronella butleri*, grow well when sucrose is used as carbon source (Kollerov et al., 2008). In the present study, we identified, cloned, and characterized an invertase GspInv from *Gongronella* sp. w5, representing the first report of invertase from this genus.

### Effects of Mono- and Di-Sugars on GspInv Activity

**FIGURE 4:**
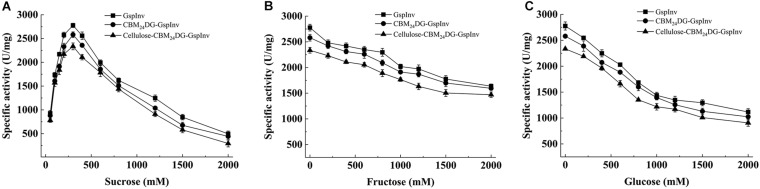
Effects of sugars on GspInv, CBM_24_DG-GspInv, and cellulose-CBM_24_DG-GspInv activities. **(A)** Sucrose. **(B)** Glucose, **(C)** Fructose. Standard deviations and values were calculated from triplicate technical repeats of measurements.

GspInv activity was determined using varying concentrations of sucrose as the substrate to evaluate the application potential of GspInv on inverted syrup and HFS production. GspInv activity increased with the increasing sucrose concentration from 10 to 300 mM. The highest invertase activity was observed at 300 mM sucrose, with a specific activity of 2,776.1 ± 124.2 U/mg protein. With the further increase in sucrose concentration, the specific activity of GspInv decreased gradually, with the *IC*_50_ (at which concentration GspInv retained 50% of the original activity) of 1.2 M. GspInv showed a specific activity of approximately 500 U/mg in the presence of 2 M sucrose (Figure 4A). Compare with sucrose, glucose and fructose inhibited GspInv activity. GspInv retained 70 and 50% of the original activities in the presence of 2 M glucose and 1.5 M fructose, respectively (Figures 4B,C).

Sucrose is regarded as one of the inhibitors that block the preparation of inverted sugar at high concentrations. Commercially used invertase from *Saccharomyces cerevisiae* is inhibited by 5% sucrose (∼146 mM) (Vásquez-Bahena et al., 2004). Another commercial yeast invertase (Bioinvert) is inhibited by 50 mM sucrose (Tomotani and Vitolo, 2007). For this reason, novel invertases tolerant to high-concentration sucrose should be explored for HFS preparation (Pérez de los Santos et al., 2016). Our data suggested the high sucrose tolerance ability of GspInv. As such, it may be a promising candidate for preparing HFS with a high sucrose concentration (>1 M). In comparison, the invertases from *A. niger* showed approximately 30% of its highest activity at 1 M sucrose (Goosen et al., 2007). Meanwhile, invertases possess high tolerance to hexoses, such as fructose and glucose, are also take advantages in HFS preparation when using alternative substrates such as molasses as the substrate. Molasses usually contains approximately 40% sucrose, 5–7% glucose, and 6–10% fructose (Najafpour and Shan, 2003). Because most invertases are inhibited by the end-product glucose and fructose (Ende and Laere, 1995; Lee and Sturm, 1996; Vorster and Botha, 1998; Hossain et al., 2011), invertases like GspInv that exhibit high hexose tolerance will facilitate the HFS preparation.

### Construction GspInv Fusion Proteins and Their Immobilization on Cellulose

**FIGURE 5:**
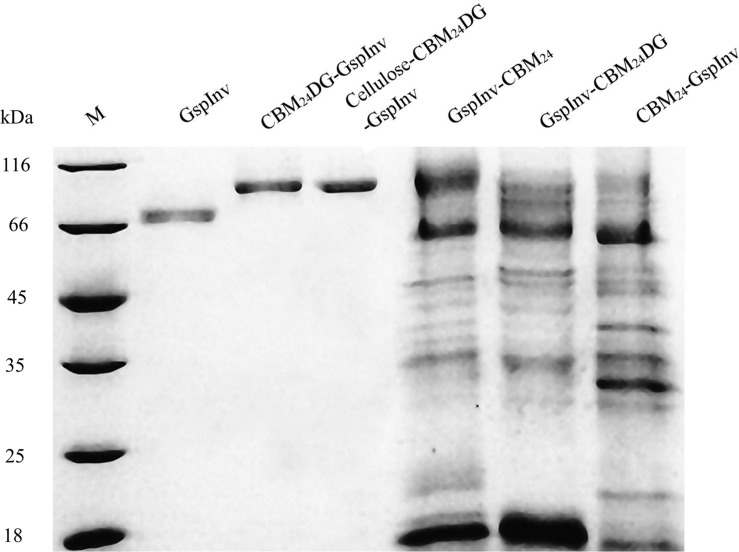
Coomassie brilliant blue R250 stained 15% SDS-PAGE gel of GspInv (fermentation broth was 10-times concentrated), CBM_24_DG-GspInv (10-times), cellulose*-*CBM_24_DG-GspInv (10-times), GspInv-CBM_24_ (500-times), GspInv-CBM_24_DG (500-times), and CBM_24_-GspInv (500-times). M: standard protein marker.

Immobilizing enzymes allow them to process large amounts of the substrate because they can be separated easily from the product; thus, the enzyme can be reused continuously. Extensive studies have been conducted on immobilizing invertases (Kotwal and Shankar, 2009). Results showed that the same kinetics could be obtained using a more conventional immobilizing matrix such as cellulose (Shoseyov and Warren, 1997; Boraston et al., 2002; Shoseyov et al., 2006; Lu et al., 2012). As a result, we immobilized GspInv on cellulose by employing CBM, without did control experiments by immobilizing GspInv on other matrixes. CBMs are small components of several cellulases that can bind specifically to cellulose (Boraston et al., 2004; Oliveira et al., 2015). Four GspInv derivates including CBM_24_-GspInv, CBM_24_DG-GspInv, GspInv-CBM_24_, and GspInv-CBM_24_DG were constructed and expressed in *K. pastoris* to facilitate the application of GspInv. Similar to GspInv, CBM_24_DG-GspInv expressed and presented as the only one band on the SDS-PAGE gel (Figure 5). Trace activity (approximately 0.4 U/mL) was detected in the culture supernatant when CBM_24_-GspInv was expressed by the strain, indicating that glycosylation may have a negative effect on CBM_24_-GspInv activity (Wan et al., 2011). No target protein bands were observed in the culture supernatants of GspInv-CBM_24_ and GspInv-CBM_24_DG (Figure 5), suggesting that CBM fused at the C-terminus of GspInv may affect the expression and/or secretion of proteins (Hussack et al., 2009; Tabuchi et al., 2010). As a result, CBM_24_DG-GspInv was chosen for further research.

**FIGURE 6:**
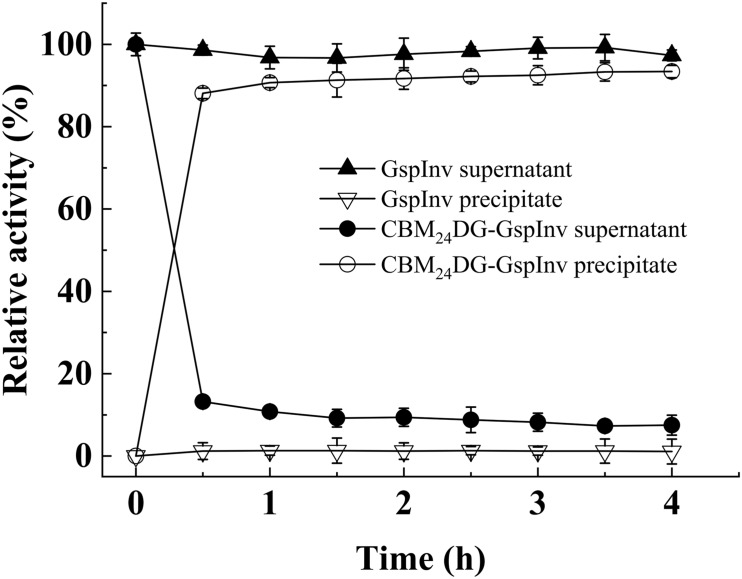
Relative invertase activities in cell-free supernatant (crude enzyme) and precipitate after adding cellulose. The total invertase activities of GspInv and CBM_24_DG-GspInv in supernatant at 0 h were set as 100% relative activity, respectively. Standard deviations and values were calculated from triplicate technical repeats of measurements.

The crude fusion enzymes were absorbed onto cellulose directly by incubating CBM_24_DG-GspInv with cellulose (designated as Cellulose-CBM_24_DG-GspInv). Only one band was observed on SDS-PAGE gel after absorbing the CBM_24_DG-GspInv onto cellulose (Figure 5). Based on the invertase activity determination, the immobilization efficiency of CBM_24_DG-GspInv reached approximately 85% after incubation with cellulose for 0.5 h and reached saturation after approximately 1 h incubation (90%). The amount of CBM_24_DG-GspInv that can adsorb to cellulose under optimal conditions was approximately 8,200 U/g cellulose, which transfer to approximately 3.5 mg of protein. The CBM_24_DG-GspInv was adsorbed irreversibly with cellulose and approximately 2% of enzyme activity (120–200 U/g cellulose) was eluted from the cellulose during washing. Meanwhile, no GspInv was bound to cellulose (Figure 6). CBMs such as CBM_24_ from family 3 usually exhibit high absorption efficiencies toward cellulose because they usually have a flat hydrophobic binding surface comprising of aromatic residues (Boraston et al., 2004; Chang et al., 2018). The planar architecture of the binding sites is consistent with the flat surfaces of crystalline polysaccharides such as cellulose, facilitating their irreversible binding and reaching a plateau within a short time (Boraston et al., 2004).

### CBM_24_DG-GspInv and Cellulose-CBM_24_DG-GspInv Characterization and Comparison

The characteristics of CBM_24_DG-GspInv and cellulose-CBM_24_DG-GspInv were determined and compared to those of GspInv. Figure 3 shows the optimum pH and temperature for CBM_24_DG-GspInv and cellulose-CBM_24_DG-GspInv were pH 5.0 and 45°C, respectively. They also shared similar pH and thermal stabilities with that of GspInv, with a half-life of approximately 100 h at 40°C and pH 5.0 (Figure 3D). Analysis of the apparent kinetic parameters showed a few differences in the *K*_m_ and *k*_cat_ values among GspInv, CBM_24_DG-GspInv, and cellulose-CBM_24_DG-GspInv. The *K*_*m*_ values of CBM_24_DG-GspInv (10.3 ± 1.2 mM) and cellulose-CBM_24_DG-GspInv (11.6 ± 1.9 mM) were slightly higher than those of GspInv (8.7 ± 1.1 mM) (Table 3). The specific activities of CBM_24_DG-GspInv (2,580 U/mg) and cellulose-CBM_24_DG-GspInv (2,335 U/mg) decreased when compared to that of GspInv (2,776U/mg) (Table 3). This phenomenon may be due to the fusion of CBM_24_ with GspInv, which caused the conformational change of the invertase and the restriction of diffusion of the substrate into the enzyme in the solid matrix (Koli and Gaikar, 2016). Thus, substrate affinity was reduced and glucose and fructose could easily enter the substrate-binding pocket.

**TABLE 3:** Kinetic parameters of GspInv, CBM_24_DG-GspInv, and Cellulose-CBM_24_DG-GspInv.

Enzyme	Specific activity (U/mg)	km (mM)	kcat (s^–1^)	kcat/km (mM^–1^s^–1^)
GspInv	2776	8.7 ± 1.1	5100 ± 10.8	595.9 ± 76.6
CBM_24_DG-GspInv	2580	10.3 ± 1.2	4588 ± 7.9	451.4 ± 52.1
Cellulose-CBM_24_DG-GspInv	2335	11.6 ± 1.9	4427 ± 11.2	393.4 ± 68.5

*Invertase activity was determined at 45°C in citrate-phosphate buffer (50 mM, pH 5.0) using sucrose (1 mM–2 M) as the substrate. The data presented are the average values from triplicate technical repeats of measurements.*

### Reusability of the Immobilized Invertase During HFS Preparation

**FIGURE 7:**
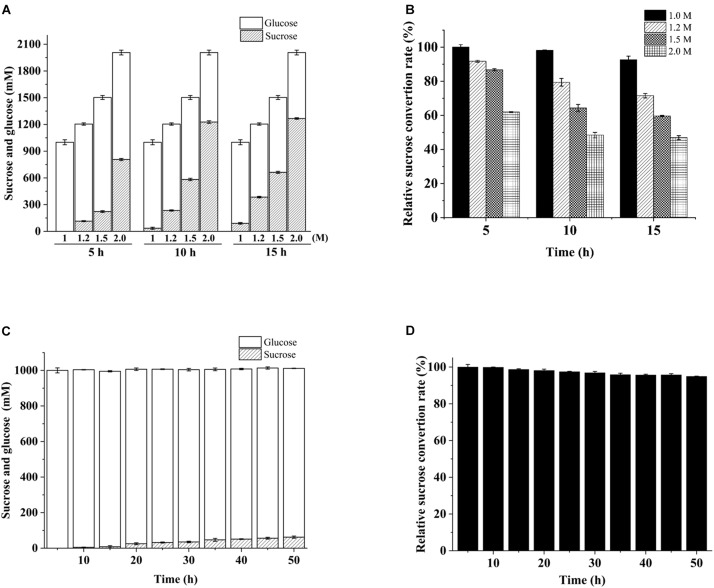
Variation in conversion during continuous substrate hydrolysis catalyzed by immobilized cellulose*-*CBM_24_DG-GspInv on PBR. **(A)** Sucrose and glucose concentration and **(B)** sucrose conversion rate after 5, 10, and 15 h continuous hydrolyzation using different concentrations of sucrose as the substrate. **(C)** Sucrose and glucose concentration and **(D)** sucrose conversion rate during 50 h continuous hydrolyzation in PBR using 1 M sucrose as the substrate. Standard deviations and values were calculated from triplicate technical repeats of measurements.

The repeated use of immobilized invertases is crucial to modern industries (Goradia et al., 2010). In our continuous flow experiment conducted on PBR, 1 M sucrose was hydrolyzed completely by cellulose-CBM_24_DG-GspInv to an equivalent amount of glucose and fructose after 15 h of reaction. In comparison, approximately 1.0–1.3 M inverted sugar was obtained in the first 5 h when 1.2–2.0 M sucrose was used as the substrate (Figure 7A). The hydrolyzation rate decreased continuously in the reaction with 1.2–2.0 M sucrose as the substrate and decreased to 50% after 15 h reaction by using 2 M sucrose as the substrate (Figure 7B). As such, sucrose at concentrations lower than 1 M was suitable for HFS preparation.

PBR containing cellulose-CBM_24_DG-GspInv was loaded continuously with 1 M sucrose for 50 h to determine further the reusability of cellulose-CBM_24_DG-GspInv. The Results showed the hydrolyzation rate was maintained at 95% after 50 h reaction (Figure 7C). Repeated use of enzymes can reduce the cost of production significantly. Several groups have reported sucrose hydrolyzation using different invertases (Table 4). Compared with such invertases, GspInv has high sucrose concentration and conversion factor, which implies its application potential in producing inverted sugars (Amaya-Delgado et al., 2006; Gómez et al., 2006; Sanjay and Sugunan, 2006; Tomotani and Vitolo, 2007; Koli and Gaikar, 2016; Oliveira et al., 2018).

**TABLE 4:** Summary of data from studies of the continuous hydrolysis of sucrose by different immobilized invertases deployed in bioreactors.

Actual source (producer)	Method of immobilization	Sucrose concentration (g/L)	Operational conditions	Conversion factor	References
GspInv (this study)	CBM bound with cellulose	342	Continuous regime in PBR, 150 mL/h 50 h, 35°C, pH5	1–0.95	This study
Bioinvert (quest internatonal)	Adsorption on polystyrene Dowex beads	0.8	Continuous regime in membrane CSTR, 20 h, 30°C, pH 5.5	1	Tomotani and Vitolo, 2007
Baker yeast (Sigma Aldrich)	Covalent binding on activated montmorillonite	100	Continuous regime in PBR, 96 h, 30°C, pH6	1–0.75	Sanjay and Sugunan, 2006
*S. cerevisiae* (Fluka)	Covalent binding on chitosan and inclusion in alginate	68.4	Continuous regime, 50 h, 30°C, pH 4.6, 20 mL/h	1–0.8	Gómez et al., 2006
*S. cerevisiae* (Fluka)	Covalent binding on nylon micro-beads	273	Continuous regime, 38 h, 50°C, pH 5.5, 318 mL/h	0.97	Amaya-Delgado et al., 2006
S. cerevisiae Bento Gonc, Brazil)	Cross-linked enzyme aggregate methodology	100	Continuous regime, 40 h, 40°C, pH 6.0, 60 mL/h	0.75	Oliveira et al., 2018
Baker yeast (Sigma Aldrich)	Invertase immobilized on glutaraldehyde crosslinked chitosan beads	170	Continuous regime, 25 h, 30°C, pH 4.0, 18 mL/h	0.96	Koli and Gaikar, 2016

Commercially, invert syrup production by using sucrose is economically unavailable due to its high price (Piotr, 2019). Molasses is considered as an alternative of sucrose because it is rich in sucrose and glucose (30–50%, v/v) and lower in price (Deng et al., 2008). The application potential of GspInv in HFS production was evaluated by using molasses as the substrate. After dilution and pretreatment, the remained liquid molasses contained 20% sucrose, 2.7% fructose, and 3.2% glucose. GspInv hydrolyzed molasses efficiently. A final concentration of 142 g/L glucose was obtained after hydrolyzing the 20% molasses with 5,000 U/L GspInv or cellulose-CBM_24_DG-GspInv for 20 min. However, quite different from that using sucrose as the substrate, the hydrolyzation rate decreased to 30% after 1 h hydrolyzation of 20% molasses on PBR containing cellulose-CBM_24_DG-GspInv and decreased to zero within 2 h. The pretreated molasses still contains substantial amounts of mineral substance (4.2% in our case) and suspended colloids. These impurities may interact with GspInv and inhibit activity continuously.

## Conclusion

In this study, GspInv from *Gongronella* sp. w5 was cloned and expressed in *K. pastoris*. Biochemical characterization indicated that GspInv is an unusual invertase with high specific activity and high tolerance to sucrose, glucose, fructose, and various metal ions. Its application potential in HFS production was explored by fusing a sequence optimized CBM to the N-terminal to purify and immobilize GspInv. The fused protein exhibited high efficiency in sucrose and molasses hydrolyzation. Our results suggest GspInv is an unusual invertase and a promising candidate for HFS preparation.

## Data Availability Statement

The sequence information of GspInv can be found in the GenBank with accession No. AVI04916.

## Author Contributions

JL and ZF conceived and designed the research. GZ, CP, XL, and FC organized and performed the experiments. JL, ZF, and YX analyzed the data and wrote the manuscript. All authors read and approved the final manuscript.

## Conflict of Interest

The authors declare that the research was conducted in the absence of any commercial or financial relationships that could be construed as a potential conflict of interest.

## Footnotes

1 https://www.uniprot.org/

2 http://smart.embl-heidelberg.de/

3 http://www.phylogeny.fr/index.cgi
