# Supplementary material for: Identification and Immobilization of an Invertase With High Specific Activity and Sucrose Tolerance Ability of Gongronella sp. w5 for High Fructose Syrup Preparation
Source: Front Microbiol. 2020 Apr 9;11:633. doi: 10.3389/fmicb.2020.00633 (PMC7160231; doi:10.3389/fmicb.2020.00633)
Supplement: Supplementary file 1 [file Data_Sheet_1.docx]

**Supplemental materials**

**Identification and immobilization of an invertase with high specific activity and sucrose tolerance ability of *Gongronella* sp. w5 for high fructose syrup preparation**

Gang Zhou^1,2,3^, Can Peng^1,2,3^, Xiaosa Liu^1,2,3^, Fei Chang^1,2,3^, Yazhong Xiao^1,2,3^, Juanjuan Liu^1,2,3*^, Zemin Fang^1,2,3*^

^1^ School of Life Sciences, Anhui University, 230601 Hefei, Anhui, China

^2^Anhui Key Laboratory of Modern Biomanufacturing, 230601 Hefei, Anhui, China

^3^Anhui Provincial Engineering Technology Research Center of Microorganisms and Biocatalysis, 230601 Hefei, Anhui, China

***Corresponding authors:** Juanjuan Liu, Zemin Fang.

Phone/Fax: +86-551-63861861

Email: liu_juan825@ahu.edu.cn (to LJ), zemin_fang@ahu.edu.cn (to FZ)

**Fig. S1 Sequence alignment of GspInv with the characterized invertases from GH32. The conserved domains and catalytic residues are shown in red boxes and triangles, respectively.**

**
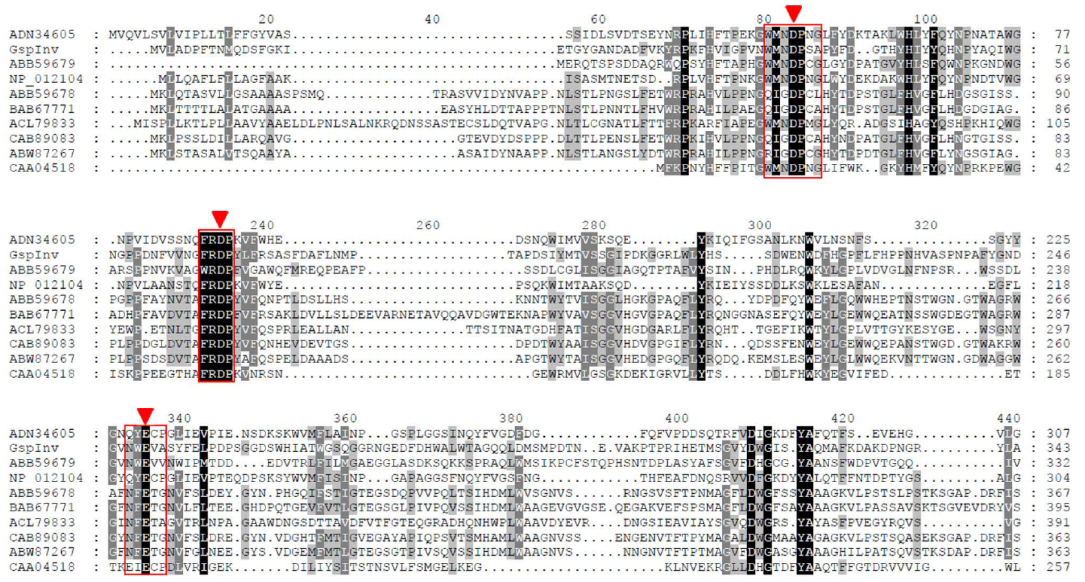
**

**Table S1 Sequence blast of GspInv with the known sequences from GenBank**

| **Strain** | **Description** | **Query coverage** | **Identity** | **E value** | **Accession No.** |
| --- | --- | --- | --- | --- | --- |
| *Absidia repens* | glycosyl hydrolase family 32 protein | 99% | 62% | 0 | ORZ22493.1 |
| *Phycomyces blakesleeanus* | [glycoside hydrolase family 32 protein](https://blast.ncbi.nlm.nih.gov/Blast.cgi#alnHdr_1069965171) | 99% | 44% | 2e-166 | [XP_018284693.1](https://www.ncbi.nlm.nih.gov/protein/1069965171?report=genbank&log$=prottop&blast_rank=1&RID=CYC8HGA9016) |
| *Basidiobolus meristosporus* | Arabinase/levansucrase/invertase | 95% | 37% | 2e-101 | OZJ06022.1 |
| [*Stachybotrys chartarum*](https://blast.ncbi.nlm.nih.gov/Blast.cgi#alnHdr_667733557) | Hypothetical protein | 91% | 32% | 2e-75 | [KFA72998.1](https://www.ncbi.nlm.nih.gov/protein/667733557?report=genbank&log$=prottop&blast_rank=2&RID=CYC8HGA9016) |
| *Stachybotrys chartarum* | Hypothetical protein | 91% | 32% | 1e-73 | [KEY67026.1](https://www.ncbi.nlm.nih.gov/protein/666401264?report=genbank&log$=prottop&blast_rank=3&RID=CYC8HGA9016) |
| *Stachybotrys chartarum* | Hypothetical protein | 91% | 32% | 5e-73 | KFA53806.1 |
| *Cryptococcus gattii* | Hypothetical protein | 88% | 33% | 1e-70 | KGB7999 |
| *Kwoniella bestiolae* | Hypothetical protein | 93% | 32% | 1e-68 | XP_019047749.1 |
| *Saitoella complicate* | Hypothetical protein | 92% | 33% | 8e-68 | GAO48875.1 |
| *Cryptococcus gattii* | Hypothetical protein | 88% | 33% | 6e-70 | KIR33250.1 |

**Table S2 MALDI-TOF-MS/MS identification of GspInv.**

| **Protein_ID** | **Protein_Qscore** | **Protein_FDR** | **Protein_Mass** | **PeptideSeqs** |  |  |
| --- | --- | --- | --- | --- | --- | --- |
| GME5616_g | 184.00397 | NA | 65368.55 | APANVKITSLNVYPITDSAFNRP | |  |
|  |  | NA | 65368.55 | DPNGRYLAVGWVQDDVIAPATSANR | |  |
|  |  | NA | 65368.55 | DPYLFR;DPYLFRSASFDAFLNMPTAPDSIYMTVSSGIPDK | | |
|  |  | NA | 65368.55 | EETVITYIPSQGNIFVNR |  |  |
|  |  | NA | 65368.55 | EETVITYIPSQGNIFVNRTASTSR | |  |
|  |  | NA | 65368.55 | ELFIQEINGIDASDPLLQEGYASWVYDASTNK | | |
|  |  | NA | 65368.55 | ELFIQEINGIDASDPLLQEGYASWVYDASTNK | | |
|  |  | NA | 65368.55 | GTGVWSLPK |  |  |
|  |  | NA | 65368.55 | GTGVWSLPKNTK |  |  |
|  |  | NA | 65368.55 | GTGVWSLPKNTKK |  |  |
|  |  | NA | 65368.55 | IETGYGANDADFVK |  |  |
|  |  | NA | 65368.55 | IETGYGANDADFVKYRPK |  |  |
|  |  | NA | 65368.55 | IHETMSGVYDWGISYAQMAFK | |  |
|  |  | NA | 65368.55 | IKPVINGPPDNFVVNGFR |  |  |
|  |  | NA | 65368.55 | IKPVINGPPDNFVVNGFRDPYLFR | |  |
|  |  | NA | 65368.55 | ITSLNVYPITDSAFNRP |  |  |
|  |  | NA | 65368.55 | IYPDDADATR |  |  |
|  |  | NA | 65368.55 | IYPDDADATRMALR |  |  |
|  |  | NA | 65368.55 | KTDLSNLAIPIDSTHVEIDAVIALDTNSDPISFVVR | | |
|  |  | NA | 65368.55 | MALRAPANVKITSLNVYPITDSAFNRP | |  |
|  |  | NA | 65368.55 | MVLADPFTNMQDSFGK |  |  |
|  |  | NA | 65368.55 | MVLADPFTNMQDSFGKIETGYGANDADFVK | | |
|  |  | NA | 65368.55 | NGEDFDHWALWTAGQQLDMSMPDTNEVAKPTPR | | |
|  |  | NA | 65368.55 | QVLAVTDDNGNSWTR |  |  |
|  |  | NA | 65368.55 | QVLAVTDDNGNSWTRIKPVINGPPDNFVVNGFR | | |
|  |  | NA | 65368.55 | SASFDAFLNMPTAPDSIYMTVSSGIPDK | |  |
|  |  | NA | 65368.55 | SASFDAFLNMPTAPDSIYMTVSSGIPDKGGR | | |
|  |  | NA | 65368.55 | SDLWRTSDETHALPLFR |  |  |
|  |  | NA | 65368.55 | TASTSRSDLWR |  |  |
|  |  | NA | 65368.55 | TDLSNLAIPIDSTHVEIDAVIALDTNSDPISFVVR | | |
|  |  | NA | 65368.55 | TLGMRPLPEYASMR |  |  |
|  |  | NA | 65368.55 | TSDETHALPLFR |  |  |
|  |  | NA | 65368.55 | TSDETHALPLFRVNNGGLNGTGGLEPLHLR | | |
|  |  | NA | 65368.55 | TTLIYTAVSYSGPTYVNGQEK |  |  |
|  |  | NA | 65368.55 | TTLIYTAVSYSGPTYVNGQEKQVLAVTDDNGNSWTR | | |
|  |  | NA | 65368.55 | VFVDNSLIEVFANDR |  |  |
|  |  | NA | 65368.55 | VFVDNSLIEVFANDRYAVSTR |  |  |
|  |  | NA | 65368.55 | VLADPFTNMQDSFGK |  |  |
|  |  | NA | 65368.55 | VNNGGLNGTGGLEPLHLR |  |  |
|  |  | NA | 65368.55 | VNNGGLNGTGGLEPLHLRVFVDNSLIEVFANDR | | |
|  |  | NA | 65368.55 | WNGVLGLYR |  |  |
|  |  | NA | 65368.55 | YAVSTRIYPDDADATR |  |  |
|  |  | NA | 65368.55 | YLAVGWVQDDVIAPATSANR |  |  |
|  |  | NA | 65368.55 | YLAVGWVQDDVIAPATSANRWNGVLGLYR | | |

**Table S3 Comparison of loss of activity of different invertases during purification. NR, not reported**

| **Enzyme** | **Fermentation activity (U/mL)** | **Specific activity in crude culture(U/mg)** | **Recovery (%)** | **References** |
| --- | --- | --- | --- | --- |
| GspInv | 147.6± 0.4 | 2337 ± 22.8 | 92 | This study |
| *Zm*INVA | NR | 854 ± 15 | 7.5 | Pérez de los Santos et.al, 2016 |
| *Zm*INVB | NR | 2278 ± 117 | 48 | Pérez de los Santos et.al, 2016 |
| *A. niger* GH1 invertase | 1.02 | NR | 18.3 | Veana et.al, 2014 |
| Ibβfruct1 | NR | 0.43 | 70.1 | Huang et.al, 2003 |
| Ibβfruct2 | 1156 | 19 | 29 | Wang et.al, 2005 |
| Ibβfruct3 | 280 | 13 | 5 | Wang et.al, 2005 |
| Boβfruct2 | NR | 58.4 | 16 | Hsieh et.al, 2006 |
| Boβfruct3 | NR | 69.5 | 23 | Hsieh et.al, 2006 |
| Suc2 | NR | 21 | 25 | Acosta et.al, 2000 |

**Table S4 Substrate specificity of GspInv towards substrates**

| **(C_6_H_10_O_5_)_n_** | **Substrate** | **Linkage** | **Specific activity (U mg^-1^)** |
| --- | --- | --- | --- |
| N=2 | Sucrose | O-α-D-glucopyranosyl-(1-2)-β-D-fructopyranoside | 2776.1±124.2 |
|  | Trehalose | O-α-D-glucopyranosyl-(1-1)-α-D-glucopyranoside | 0 |
|  | Cellobiose | O-β-D-glucopyranosyl-(1-4)-D-glucopyranoside | 0 |
|  | Maltose | O-α-D-glucopyranosyl-(1-4)-D-glucopyranoside | 0 |
|  | Isomaltose | O-α-D-glucopyranosyl-(1-6)-D-glucopyranoside | 0 |
| N=3 | Raffinose | O-α-D- galactopyranosyl -(1-6)-D- glucopyranosyl -(1-2)-β-D- fructopyranoside | 2098.7±123.6 |
|  | Melizitose | O-α-D- glucopyranosyl -(1-6)-D- fructofuranosyl -(1-4)-D- glucopyranoside | 0 |
| N=4 | Stachyose | O-α-D-fructofuranosyl-(1-6)-D-galactopyranosyl-(1-6)-D-galactopyranosyl-(1-6)-D-glucopyranoside | 0 |
| N>20 | Inulin | O-β-D-fructofuranosyl-(2-1)-[D-galactopyranosyl]n--D-glucopyranoside | 0 |
